# Giant Cell Tumour Presenting with Chronic Ankle-Joint Pain

**DOI:** 10.31138/mjr.33.4.453

**Published:** 2022-12-31

**Authors:** Christina Flourou, Andreas Tofarides, Eleni Papanicolaou, Andreas Liampas, Savvas Psarelis

**Affiliations:** 1 Internal Medicine Department, Nicosia General Hospital, Nicosia, Cyprus,; 2 Aristotle University of Thessaloniki, Thessaloniki, Greece,; 3 Rheumatology Department, Nicosia General Hospital, Nicosia, Cyprus

**Keywords:** giant cell tumour, chronic pain, ankle-joint

A 62-year-old patient, non-smoker, with prior medical history of hypertension was referred to Rheumatology. The patient described pain and right (RT) ankle-joint swelling. He mentioned a gradually progressive pain in his joints, that amplified with exercise. He also denied any recent trauma or overuse. He had no fever, chills, sweats, or other constitutional symptoms.

Following a physical examination revealed swelling of ankle-joint, moderate pain on palpation, without redness or warmth difference between his left (LT) ankle-joint. Metatarsal joints were not affected and no other significant signs from musculoskeletal system were observed. X-ray imaging of the RT foot did not reveal any signs of fractures, osteoarthritis or chondrocalcinosis and no joint effusion was presented.

The laboratory workup was unremarkable with normal inflammatory markers, normal RF, uric acid, and no autoantibodies. Serological test came back normal.

Ultrasound sonography revealed a heterogeneous mass on the outer front portion of the ankle (**Figure 1A**). Further Magnetic Resonance Imaging confirmed the presence of a solid heterogenous mass with dimensions 1.6x2.5x2.8cm (**Figure 1B**).

**Figure 1.**
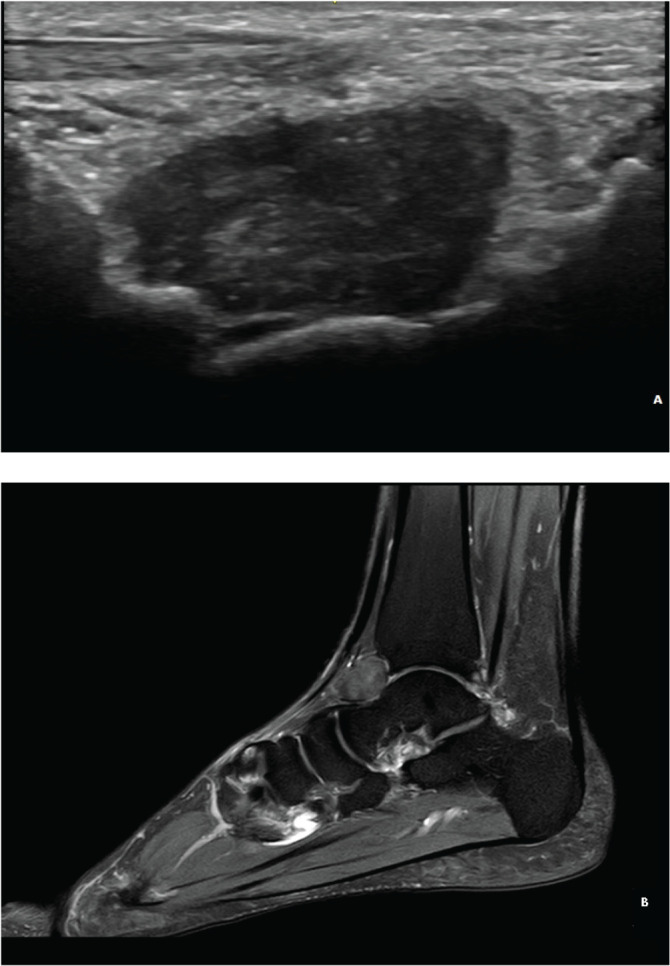
Ultrasound Sonography of the RT ankle-joint showing heterogeneous lesion with irregular margin to the outer front portion of the ankle (A). Magnetic Resonance Imaging scan (T1-W) demonstrating a solid heterogeneous lesion with dimensions 1.6x2.5x2.8cm (B).

Over the next weeks, surgical resection of the mass was performed as to undergo further histopathological analysis. The microscopical examination confirmed the diagnosis of a classic benign giant cell tumour of the tendon sheath. The tumour presented a small hyperchromatic fused oval shaped cells with several large osteoclasts such as multinucleated giant cells scattered throughout with few dilated vessels and oedema but no cellular atypia (**Figure 2**).

**Figure 2.**
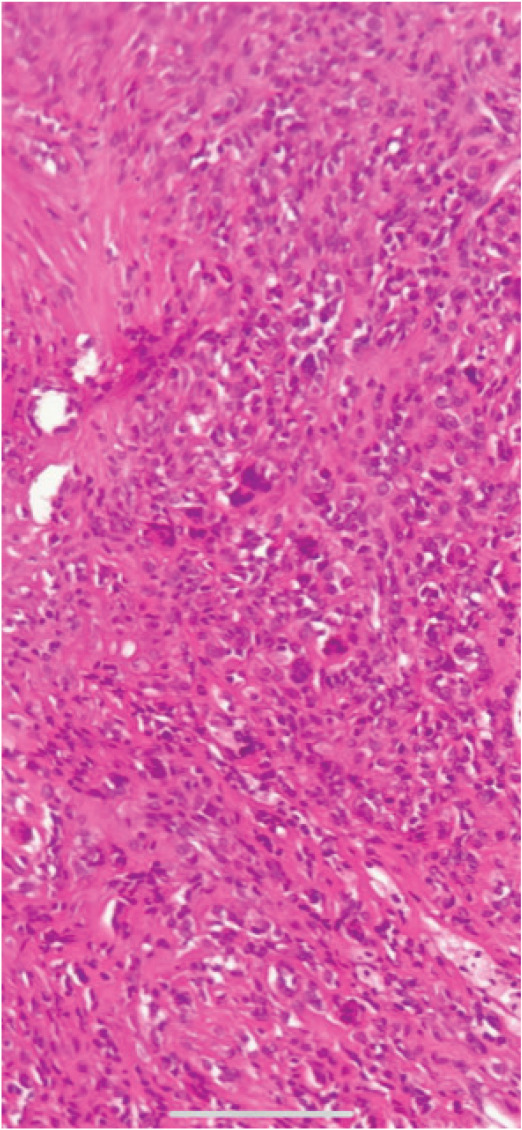
Left eye fundus photograph of posterior pole showing numerous flame-shaped superficial retinal haemorrhages, some with white centre (Roth spot) and peri-papillary subretinal haemorrhages.

The patient recovered fully within few weeks. Tenosynovial giant cell tumours are rare^1^ lesion arising from synovia of joints or tendon sheaths. They always involve a single joint: most commonly, the knee or ankle joint limiting joint function and destroying the adjacent bones.^3^ The principal treatment is tumour resection and limited data support radiation therapy due to the risk of long-term toxicity.^4^

This case is useful to remind that joint involvement with chronic pain (over the six-week duration) required further evaluation with imaging tools when osteoarthritis exacerbation, crystal arthropathy, infectious and systemic rheumatic diseases are carefully ruled out.

## ABBREVIATIONS

LT: Left
RT: Right
